# Supplementary material for: Disproportionate CH4 Sink Strength from an Endemic, Sub-Alpine Australian Soil Microbial Community
Source: Microorganisms. 2021 Mar 15;9(3):606. doi: 10.3390/microorganisms9030606 (PMC8002156; doi:10.3390/microorganisms9030606)
Supplement: Supplementary file 1 [file microorganisms-09-00606-s001.pdf]

Supplementary Information

Disproportionate CH_4 sink strength from an endemic, sub-alpine Australian soil microbial community

M.D. McDaniel[1,2*‡], M. Hernández[3,4*], M.G. Dumont[3,5], L.J. Ingram[1], and M.A. Adams[1,6]

1. Centre for Carbon Water and Food | Sydney Institute of Agriculture | University of Sydney | Sydney, Australia 2000
2. Department of Agronomy | Iowa State University | Ames, Iowa USA 50011
3. Max Planck Institute for Terrestrial Microbiology | Marburg, Germany D-35037
4. School of Environmental Sciences | Norwich Research Park | University of East Anglia, Norwich, UK NR4 7TJ
5. Centre for Biological Sciences | University of Southampton | Southampton, UK SO17 1BJ
6. School of Science | Engineering and Technology | The University of Swinburne | Melbourne, Australia 3122

Supplementary Information Table of Contents

TABLES

Table S1. Chamber and soil microclimate at time of sampling, and incubation temperature by date in 2015 (means ± standard errors)† 3

Table S2. Dynamic soil physical and chemical characteristics by date in 2015 (means ± standard errors)†....4

Table S3. Static soil physical and chemical characteristics – from 17 February 2015 (means ± standard errors)....................6

Table S4. Concentrations of heavy elements in soils based on Niton XL3t Ultra Analyzer meter – from 17 February 2015 (means ± standard errors) 7

Table S5. Barcode identification for each of the samples analyzed. Raw data were deposited under the study accession number PRJNA384296 in the NCBI Sequence Read Archive (SRA) 8

FIGURES

Figure S1. Bacterial 16S rRNA gene diversity measured as Shannon Diversity (H') and Richness (S) (n=4, means ± standard errors). 10

Figure S2. NMDS ordination of *pmoA* (A) and euryarchaeota (B) communities based on the Bray–Curtis dissimilarity of community composition. Shape indicates depth and sites are colored according to the soil typel. The arrows indicate the direction at which the environmental vectors fit the best (using the *envfit* function) onto the NMDS ordination space. Abbreviations: DOC, dissolved organic carbon; DON, dissolved organic nitrogen; EC, electrical conductivity; GWC, gravimetric water content; NH_4, ammonium. 12

Figure S3. Example of sampling locations and streams located in a High Country region near Mt. Kosciuszko National Park, NSW, Australia. Soil/vegetation types shown along one transect in the picture above. Below are three soil cores from each of the soil/vegetation types. 13

Figure S4. Areal coverage of Australian Alps in southeastern Australia (1.2M ha). 14

Table S1. Chamber and soil microclimate at time of sampling, and incubation temperature by date in 2015 (means ± standard errors)[†]

Date	Soil-Vegetation Type	GHG Chamber Temperature	Soil Temperature	Incubation Room Temperature Range‡	Volumetric Moisture Content
		--------------------- º C	---------------------		--- m^3 m^{-3} ---
February 17th †	Forest	20.5 ± 1.9	13.4 ± 0.2	20.9 to 22.5	26.7 ± 4.9
	Grassland	28.3 ± 2.1	16.4 ± 0.8		22.2 ± 1.8
	Bog	27.2 ± 3.2	14.1 ± 0.6		68.3 ± 5.7
May 25th	Forest	10.1 ± 0.9	3.9 ± 0.4	4.9 to 5.6	18.4 ± 2.3
	Grassland	7.5 ± 2.3	3.6 ± 0.2		27.2 ± 0.6
	Bog	7.5 ± 1.4	3.6 ± 0.8		77.2 ± 9.1
September 22nd	Forest	4.1 ± 0.7	4.3 ± 0.4	5.0 to 5.7	28.3 ± 3
	Grassland	5.1 ± 1	4 ± 0.3		27.1 ± 1.7
	Bog	3.1 ± 2.4	4.6 ± 0.3		85.7 ± 9.3
November 23rd	Forest	21 ± 1.7	12.7 ± 1.1	13.8 to 15.6	10.5 ± 2.2
	Grassland	17.8 ± 1.8	13 ± 1.1		14.1 ± 1.6
	Bog	16.3 ± 1.9	11.5 ± 0.8		57.5 ± 9.6

†: Date of soil microbial community analyses.

‡: Intended to correspond to field conditions as close as possible

Table S2. Dynamic soil physical and chemical characteristics by date in 2015 (means ± standard errors)†

Date	Soil Type	Depth (cm)	Gravimetric Water Content	Ammonium	Nitrate
			---- $g\ g^{-1}$ ----	---------------- $mg\ kg^{-1}$ ---------------	
February 17th ‡	Forest	0 – 5	0.79 ± 0.1	73.08 ± 17.87	7.57 ± 7.27
		5 – 10	0.57 ± 0.2	63.92 ± 31.45	3.66 ± 3.41
		10 – 15	0.52 ± 0.18	49.26 ± 22.93	1.55 ± 1.37
		15 – 20	0.43 ± 0.13	39.62 ± 15.13	1.15 ± 0.8
		20 – 25	0.43 ± 0.14	35.12 ± 17.22	0.71 ± 0.47
		25 – 30	0.25 ± 0.02	16.94 ± 1.81	0.41 ± 0.16
	Grassland	0 – 5	0.38 ± 0.06	37.69 ± 4.54	0.75 ± 0.29
		5 – 10	0.34 ± 0.03	26.08 ± 1.81	0.47 ± 0.1
		10 – 15	0.31 ± 0.03	19.49 ± 1.73	0.54 ± 0.13
		15 – 20	0.28 ± 0.03	15.09 ± 1	0.43 ± 0.06
		20 – 25	0.29 ± 0.05	13.41 ± 0.85	0.45 ± 0.07
		25 – 30	0.25 ± 0.03	11.21 ± 0.48	0.58 ± 0.04
	Bog	0 – 5	4.66 ± 0.92	205.81 ± 21.75	0.61 ± 0.1
		5 – 10	5.83 ± 3.45	160.3 ± 34.53	0.29 ± 0.06
		10 – 15	2.29 ± 0.9	82.27 ± 23.49	0.21 ± 0.01
		15 – 20	1.16 ± 0.23	38.18 ± 11.53	0.27 ± 0.05
		20 – 25	1.36 ± 0.43	32.06 ± 11.11	0.2 ± 0.03
		25 – 30	0.93 ± 0.15	19.35 ± 5.92	0.16 ± 0.03
May 25th	Forest	0 – 5	0.8 ± 0.19	3.17 ± 0.8	0.21 ± 0.08
		5 – 10	0.46 ± 0.07	3.47 ± 0.43	0.18 ± 0.11
		10 – 15	0.46 ± 0.1	4.31 ± 1.18	0.25 ± 0.1
		15 – 20	0.42 ± 0.09	3.53 ± 0.26	0.16 ± 0.07
		20 – 25	0.42 ± 0.1	2.83 ± 0.38	0.17 ± 0.06
		25 – 30	0.32 ± 0.05	2.86 ± 0.31	0.22 ± 0.12
	Grassland	0 – 5	0.43 ± 0.04	60.31 ± 3.72	BDL
		5 – 10	0.37 ± 0.02	40.78 ± 0.45	1.28 ± 0.83
		10 – 15	0.35 ± 0.02	30.68 ± 1.63	0.71 ± 0.15
		15 – 20	0.34 ± 0.02	23.75 ± 0.83	0.15 ± 0.04
		20 – 25	0.32 ± 0.02	19.47 ± 1.18	BDL
		25 – 30	0.31 ± 0.02	17.7 ± 2.06	0.29 ± 0.12
	Bog	0 – 5	8.7 ± 5.21	109.63 ± 46.63	0.49 ± 0.03
		5 – 10	3.58 ± 1.78	70.63 ± 35.02	0.87 ± 0.28
		10 – 15	4.75 ± 2.77	98.8 ± 16.42	0.42 ± 0.14
		15 – 20	12.5 ± 9.84	36.69 ± 0	0.94 ± 0.01
		20 – 25	1.55 ± 0.33	36.65 ± 2.59	BDL
		25 – 30	1.06 ± 0.27	7.29 ± 4.57	0.24 ± 0.1
September 22nd	Forest	0 – 5	0.91 ± 0.25	126.08 ± 10.56	2.14 ± 1.55
		5 – 10	0.56 ± 0.14	67.03 ± 10.03	1.46 ± 1
		10 – 15	0.52 ± 0.13	54.36 ± 9.17	0.9 ± 0.56
		15 – 20	0.47 ± 0.09	58.92 ± 14.93	0.8 ± 0.5
		20 – 25	0.44 ± 0.08	51.63 ± 14.74	0.48 ± 0.22
		25 – 30	0.42 ± 0.11	35.61 ± 10.14	0.4 ± 0.09
	Grassland	0 – 5	0.45 ± 0.03	94.52 ± 7.72	0.71 ± 0.24
		5 – 10	0.36 ± 0.02	87.32 ± 24.64	0.83 ± 0.14
		10 – 15	0.34 ± 0.02	37.95 ± 5.23	0.85 ± 0.24
		15 – 20	0.32 ± 0.02	26.73 ± 3.46	1.01 ± 0.12
		20 – 25	0.3 ± 0.02	19.89 ± 2.6	0.86 ± 0.14
		25 – 30	0.29 ± 0.02	17.9 ± 1.23	1.03 ± 0.11
	Bog	0 – 5	4.93 ± 1.34	152.3 ± 6.16	0.26 ± 0.03
		5 – 10	1.91 ± 0.25	128.86 ± 30.83	0.28 ± 0.03
		10 – 15	1.12 ± 0.22	73.28 ± 28.6	0.19 ± 0.01
		15 – 20	1.24 ± 0.23	85.1 ± 29.85	0.23 ± 0
		20 – 25	1.3 ± 0.06	78 ± 12.68	0.18 ± 0.03

		25 – 30	0.94 ± 0.03	42.34 ± 4.62	BDL
November 23rd	Forest	0 – 5	0.55 ± 0.06	140.41 ± 16.54	BDL
		5 – 10	0.4 ± 0.06	76.66 ± 13.65	BDL
		10 – 15	0.34 ± 0.06	61.67 ± 9.83	BDL
		15 – 20	0.3 ± 0.04	47.32 ± 7.09	BDL
		20 – 25	0.26 ± 0.03	36.05 ± 1.91	BDL
		25 – 30	0.25 ± 0.01	21.73 ± 0.37	BDL
	Grassland	0 – 5	0.26 ± 0.04	76.44 ± 7.66	BDL
		5 – 10	0.29 ± 0.02	64.11 ± 6.2	BDL
		10 – 15	0.29 ± 0.02	47.18 ± 4.03	BDL
		15 – 20	0.29 ± 0.02	35.76 ± 3.38	BDL
		20 – 25	0.28 ± 0.03	32.34 ± 2.45	BDL
		25 – 30	0.27 ± 0.03	26.16 ± 1.6	BDL
	Bog	0 – 5	3.87 ± 1.53	224.65 ± 6.23	0.41 ± 0.13
		5 – 10	3.19 ± 1.28	223 ± 42.37	BDL
		10 – 15	2.35 ± 0.81	191.28 ± 52.98	BDL
		15 – 20	1.6 ± 0.61	90.36 ± 27.71	BDL
		20 – 25	0.95 ± 0.25	30.89 ± 8.46	BDL
		25 – 30	0.68 ± 0.15	ND	ND

†: BDL, soil extract below detection limit (0.03 mg L^{-1}); ND, no data
‡: Date of soil microbial community analyses.

Table S3. Static soil physical and chemical characteristics – from 17 February 2015 (means ± standard errors)

Soil Type	Depth (cm)	Sand	Silt	Clay	Gravel & Rocks	Roots or Rhizoids	pH	Electrical Conductivity	Total Organic Carbon	Total Nitrogen
		-------------- % --------------			---------- *g cm*$^{-3}$ ---------		*unitless*	*µS cm*$^{-1}$	-------------- % --------------	
Forest	0 – 5	74 ± 0	9 ± 1	17 ± 1	64 ± 20	9.68 ± 1.24	5.46 ± 0.14	163 ± 44	10.11 ± 1.30	0.58 ± 0.08
	5 – 10	73 ± 1	12 ± 1	15 ± 1	88 ± 33	2.05 ± 0.69	5.45 ± 0.22	72 ± 19	7.90 ± 1.93	0.44 ± 0.12
	10 – 15	72 ± 3	13 ± 2	14 ± 1	114 ± 34	3.06 ± 1.18	5.50 ± 0.14	65 ± 19	5.46 ± 0.97	0.31 ± 0.07
	15 – 20	76 ± 2	9 ± 1	15 ± 1	90 ± 16	1.42 ± 0.73	5.51 ± 0.15	47 ± 11	4.88 ± 1.12	0.27 ± 0.08
	20 – 25	72 ± 2	13 ± 3	14 ± 2	100 ± 19	3.67 ± 1.01	5.53 ± 0.10	45 ± 12	4.63 ± 1.11	0.25 ± 0.07
	25 – 30	68 ± 2	16 ± 2	16 ± 2	134 ± 26	1.30 ± 0.86	5.55 ± 0.09	40 ± 14	4.11 ± 1.06	0.22 ± 0.06
Grassland	0 – 5	64 ± 3	15 ± 2	22 ± 2	17 ± 4	5.35 ± 1.27	5.54 ± 0.08	118 ± 34	5.97 ± 0.42	0.41 ± 0.03
	5 – 10	55 ± 3	20 ± 1	24 ± 3	20 ± 5	0.28 ± 0.13	5.54 ± 0.06	55 ± 6	4.67 ± 0.19	0.32 ± 0.01
	10 – 15	56 ± 2	20 ± 2	24 ± 2	11 ± 3	0.22 ± 0.12	5.49 ± 0.05	41 ± 5	3.86 ± 0.11	0.26 ± 0.01
	15 – 20	53 ± 2	21 ± 3	26 ± 2	13 ± 3	0.09 ± 0.03	5.52 ± 0.04	31 ± 3	3.1 ± 0.19	0.21 ± 0.01
	20 – 25	54 ± 3	20 ± 3	25 ± 2	17 ± 4	1.21 ± 1.12	5.52 ± 0.06	24 ± 2	2.61 ± 0.17	0.18 ± 0.01
	25 – 30	55 ± 3	20 ± 3	24 ± 1	22 ± 3	0.05 ± 0.04	5.58 ± 0.08	24 ± 3	2.34 ± 0.22	0.16 ± 0.01
Bog	0 – 5	60 ± 6	23 ± 6	17 ± 0	7 ± 3	12.41 ± 2.03	5.38 ± 0.10	488 ± 62	12.78 ± 0.96	0.69 ± 0.07
	5 – 10	75 ± 2	19 ± 2	6 ± 1	121 ± 67	3.79 ± 1.16	5.51 ± 0.04	91 ± 19	9.59 ± 4.26	0.54 ± 0.18
	10 – 15	80 ± 2	14 ± 3	6 ± 1	72 ± 44	1.01 ± 0.19	5.58 ± 0.02	40 ± 6	11.47 ± 3.27	0.72 ± 0.14
	15 – 20	79 ± 3	15 ± 2	6 ± 1	119 ± 57	1.30 ± 0.26	5.53 ± 0.00	36 ± 4	8.29 ± 2.67	0.5 ± 0.15
	20 – 25	82 ± 1	11 ± 2	7 ± 0	203 ± 63	2.52 ± 1.07	5.63 ± 0.02	27 ± 3	5.82 ± 0.76	0.34 ± 0.07
	25 – 30	82 ± 5	11 ± 3	7 ± 2	129 ± 46	2.05 ± 0.58	5.69 ± 0.02	20 ± 5	3.72 ± 0.72	0.2 ± 0.04

Table S4. Concentrations of heavy elements in soils based on Niton XL3t Ultra Analyzer – from 17 February 2015 (means ± standard errors)

Soil Type	Depth (cm)	Al	Fe	K	Si	As	Ba	Bi	Ca	Cr	Mg	Mn	Nb	P	Pb	Rb	S	Sr	Ti	V	Zn	Zr
		------------- $g\ kg^{-1}$ -------------				------------------------ $mg\ kg^{-1}$ ------------------------																
Forest	0 – 5	33.4 ± 4.9	26.4 ± 1.4	11.6 ± 1.3	142.7 ± 5.3	8 ± 1	114 ± 11	9 ± 0	9279 ± 1929	76 ± 6	4460 ± 109	2125 ± 409	13 ± 1	1808 ± 210	19 ± 7	74 ± 8	2738 ± 499	75 ± 13	2821 ± 112	102 ± 7	47 ± 8	164 ± 5
	5 – 10	45.5 ± 5.6	30.8 ± 1	13.5 ± 1.4	1jh 70.1 ± 6.9	10 ± 1	231 ± 17	13 ± 2	2710 ± 715	95 ± 14	ND	1474 ± 410	14 ± 1	1442 ± 177	17 ± 4	90 ± 5	1486 ± 265	59 ± 7	3281 ± 151	122 ± 13	49 ± 6	209 ± 28
	10 – 15	47.8 ± 7.2	32 ± 1	13.6 ± 1.3	159 ± 10.2	7 ± 0	236 ± 11	14 ± 2	4663 ± 2052	128 ± 6	5689 ± 543	1244 ± 275	14 ± 1	1625 ± 254	26 ± 10	94 ± 3	1542 ± 250	64 ± 8	3497 ± 106	138 ± 4	53 ± 11	238 ± 53
	15 – 20	53.9 ± 4.3	32.8 ± 1.3	15.4 ± 1.2	175 ± 12.9	12 ± 1	242 ± 35	16 ± 1	2445 ± 1022	120 ± 11	3875 ± 386	713 ± 69	14 ± 1	1493 ± 288	19 ± 6	99 ± 7	1025 ± 106	59 ± 4	3635 ± 288	129 ± 9	44 ± 8	214 ± 39
	20 – 25	65.2 ± 3.1	32.4 ± 0.2	15.7 ± 0.3	178.4 ± 8.8	12 ± 0	279 ± 16	19 ± 1	1641 ± 431	106 ± 6	3120 ± 209	502 ± 136	15 ± 1	1024 ± 45	27 ± 13	91 ± 1	775 ± 148	47 ± 3	3496 ± 47	135 ± 6	40 ± 7	179 ± 12
	25 – 30	67.3 ± 2.8	33.4 ± 0.8	15.9 ± 0.8	170.1 ± 9.2	13 ± 1	276 ± 9	18 ± 3	1392 ± 320	101 ± 11	3675 ± 839	382 ± 102	13 ± 1	934 ± 82	21 ± 9	98 ± 2	650 ± 117	49 ± 4	3293 ± 90	132 ± 7	42 ± 13	174 ± 12
Grassland	0 – 5	50.3 ± 2.9	31.9 ± 2.8	14.7 ± 1.5	176 ± 11	9 ± 1	129 ± 17	12 ± 2	1784 ± 490	99 ± 11	ND	769 ± 237	12 ± 1	1268 ± 105	21 ± 4	85 ± 9	1788 ± 198	36 ± 1	3205 ± 446	131 ± 17	56 ± 9	194 ± 21
	5 – 10	58.5 ± 2.1	34.4 ± 3.2	15.2 ± 1.3	184.6 ± 11.2	8 ± 0	164 ± 6	16 ± 1	906 ± 242	115 ± 15	5076 ± 987	668 ± 230	14 ± 2	1126 ± 111	25 ± 4	93 ± 8	1253 ± 89	35 ± 1	3440 ± 571	139 ± 15	51 ± 7	198 ± 36
	10 – 15	61.6 ± 1.4	34.8 ± 2.8	15.2 ± 1.3	186.2 ± 11	ND	170 ± 5	18 ± 1	755 ± 186	106 ± 11	4023 ± 438	613 ± 248	15 ± 2	1037 ± 82	24 ± 3	95 ± 9	1095 ± 37	34 ± 2	3494 ± 564	135 ± 18	46 ± 8	204 ± 36
	15 – 20	65.1 ± 2.8	34.2 ± 3.3	15.8 ± 1.5	189.7 ± 7.9	ND	172 ± 7	18 ± 2	593 ± 113	122 ± 17	ND	526 ± 183	15 ± 2	1010 ± 127	27 ± 6	96 ± 9	936 ± 49	33 ± 2	3445 ± 538	139 ± 12	50 ± 7	204 ± 44
	20 – 25	70.5 ± 4.2	34.3 ± 2.8	16.3 ± 2.1	193.8 ± 9.1	9 ± 0	201 ± 15	22 ± 1	503 ± 100	109 ± 13	3629 ± 107	432 ± 154	14 ± 1	878 ± 63	25 ± 5	95 ± 11	820 ± 29	33 ± 1	3547 ± 452	136 ± 15	46 ± 5	201 ± 34
	25 – 30	77.8 ± 4.4	35.2 ± 3.4	18 ± 2.3	194.5 ± 9.1	9 ± 0	215 ± 2	21 ± 2	471 ± 56	141 ± 10	3406 ± 418	449 ± 166	14 ± 2	916 ± 125	25 ± 5	97 ± 11	802 ± 19	32 ± 1	3813 ± 500	152 ± 22	44 ± 5	211 ± 34
Bog	0 – 5	25.8 ± 3	26.9 ± 3.5	10.1 ± 0.6	183.4 ± 11.4	ND	ND	ND	5572 ± 350	88 ± 13	4774	1152 ± 426	14 ± 1	1894 ± 107	13 ± 0	53 ± 2	3653 ± 56	62 ± 3	2661 ± 85	110 ± 5	109 ± 46	135 ± 6
	5 – 10	30.4 ± 1.9	24.7 ± 3.2	11.4 ± 0.9	218.3 ± 21.7	9 ± 1	114 ± 4	8 ± 0	2281 ± 396	93 ± 6	4208 ± 0	643 ± ND	15 ± 1	1701 ± 104	11 ± 1	65 ± 3	2131 ± 187	49 ± 4	3036 ± 71	117 ± 9	49 ± 10	250 ± 11
	10 – 15	32.5 ± 1.1	24.4 ± 4.1	13.2 ± 0.7	231.2 ± 23	12 ± 1	178 ± 15	13 ± 1	1578 ± 361	95 ± 10	3246 ± 448	261 ± ND	14 ± 3	1481 ± 44	10 ± 0	69 ± 4	1492 ± 62	40 ± 6	3049 ± 290	110 ± 16	32 ± 6	238 ± 40
	15 – 20	40.4 ± 4.3	24.1 ± 3.9	17.4 ± 2.2	239.3 ± 5.7	8 ± 1	282 ± 85	15 ± 1	1180 ± 306	93 ± 23	4828 ± ND	314 ± ND	14 ± 3	1288 ± 129	17 ± 4	82 ± 9	1199 ± 151	35 ± 6	3330 ± 291	119 ± 23	33 ± 10	264 ± 47
	20 – 25	40 ± 3.8	20.7 ± 3.2	16.5 ± 2	248.1 ± 10	9 ± ND	286 ± 68	13 ± 2	1094 ± 300	83 ± 6	5256 ± 1925	179 ± ND	14 ± 2	1202 ± 94	17 ± 2	75 ± 6	1211 ± 224	34 ± 7	3193 ± 276	108 ± 16	31 ± 8	258 ± 32
	25 – 30	42.9 ± 4.5	22.4 ± 4	18.2 ± 2.6	256.3 ± 10.3	ND	313 ± 99	16 ± 1	1077 ± 291	97 ± 25	5370 ± 801	279 ± ND	11 ± 3	961 ± 116	16 ± 3	82 ± 11	1027 ± 222	30 ± 7	3325 ± 378	119 ± 13	32 ± 9	176 ± 37

ND = no data, either when no values or only one value making standard error impossible to calculate

Table S5. Barcode identification for each of the samples analyzed. Raw data were deposited under the study accession number PRJNA384296 in the NCBI Sequence Read Archive (SRA). For 16S rRNA genes, primers used: F515 (5'-GTGCCAGCMGCCGCGGTAA-3'), R806 (5'-GGACTACVSGGGTATCTAAT-3'). For *pmoA* genes, primer set first round PCR (A189f/A682r) and second round multiplex PCR (A189f/A650r/mb661r): A189f (5'-GGNGACTGGGACTTCTGG-3'), A682r (5'-GAASGCNGAGAAGAASGC-3'), A650r (5'-ACGTCCTTACCGAAGGT-3'), mb661r (5'-CCGGMGCAACGTCYTTACC-3')

Sample	Target gene	Soil – file name	Barcode
B1-0	16S rRNA	fresh soil-bog- depth0cm-replicate-1	GTCACA
B2-0	16S rRNA	fresh soil-bog- depth0cm-replicate-2	TAGCAT
B3-0	16S rRNA	fresh soil-bog- depth0cm-replicate-3	ACGTAC
B4-0	16S rRNA	fresh soil-bog- depth0cm-replicate-4	TCAGAG
B1-5	16S rRNA	fresh soil-bog- depth5cm-replicate-1	AGCTGA
B2-5	16S rRNA	fresh soil-bog- depth5cm-replicate-2	CACAGT
B3-5	16S rRNA	fresh soil-bog- depth5cm-replicate-3	AGAGTC
B4-5	16S rRNA	fresh soil-bog- depth5cm-replicate-4	CGTATA
B1-10	16S rRNA	fresh soil-bog- depth10cm-replicate-1	AGTCAG
B2-10	16S rRNA	fresh soil-bog- depth10cm-replicate-2	CAGTCA
B3-10	16S rRNA	fresh soil-bog- depth10cm-replicate-3	AGCTGA
B4-10	16S rRNA	fresh soil-bog- depth10cm-replicate-4	GACTAG
B1-15	16S rRNA	fresh soil-bog- depth15cm-replicate-1	ATATCG
B2-15	16S rRNA	fresh soil-bog- depth15cm-replicate-2	CATGAC
B3-15	16S rRNA	fresh soil-bog- depth15cm-replicate-3	AGTCAG
B4-15	16S rRNA	fresh soil-bog- depth15cm-replicate-4	GAGATC
B1-20	16S rRNA	fresh soil-bog- depth20cm-replicate-1	ATCGAT
B2-20	16S rRNA	fresh soil-bog- depth20cm-replicate-2	CGATAT
B3-20	16S rRNA	fresh soil-bog- depth20cm-replicate-3	ATATCG
B4-20	16S rRNA	fresh soil-bog- depth20cm-replicate-4	GATCGA
B1-25	16S rRNA	fresh soil-bog- depth25cm-replicate-1	ATGCTA
B2-25	16S rRNA	fresh soil-bog- depth25cm-replicate-2	CGCGCG
B3-25	16S rRNA	fresh soil-bog- depth25cm-replicate-3	ATCGAT
B4-25	16S rRNA	fresh soil-bog- depth25cm-replicate-4	GTACAC
F1-0	16S rRNA	fresh soil-forest- depth0cm-replicate-1	TACGTA
F2-0	16S rRNA	fresh soil-forest- depth0cm-replicate-2	TATACG
F3-0	16S rRNA	fresh soil-forest- depth0cm-replicate-3	ACTGCA
F4-0	16S rRNA	fresh soil-forest- depth0cm-replicate-4	TCTCTC
F1-5	16S rRNA	fresh soil-forest- depth5cm-replicate-1	ACACGT
F2-5	16S rRNA	fresh soil-forest- depth5cm-replicate-2	AGTCAG
F3-5	16S rRNA	fresh soil-forest- depth5cm-replicate-3	CGTATA
F4-5	16S rRNA	fresh soil-forest- depth5cm-replicate-4	CAGTCA
F1-10	16S rRNA	fresh soil-forest- depth10cm-replicate-1	ACGTAC
F2-10	16S rRNA	fresh soil-forest- depth10cm-replicate-2	ATATCG
F3-10	16S rRNA	fresh soil-forest- depth10cm-replicate-3	GACTAG
F4-10	16S rRNA	fresh soil-forest- depth10cm-replicate-4	CATGAC
F1-15	16S rRNA	fresh soil-forest- depth15cm-replicate-1	ACTGCA
F2-15	16S rRNA	fresh soil-forest- depth15cm-replicate-2	ATCGAT
F3-15	16S rRNA	fresh soil-forest- depth15cm-replicate-3	GAGATC

F4-15	16S rRNA	fresh soil-forest- depth15cm-replicate-4	CGATAT
F1-20	16S rRNA	fresh soil-forest- depth20cm-replicate-1	AGAGTC
F2-20	16S rRNA	fresh soil-forest- depth20cm-replicate-2	ATGCTA
F3-20	16S rRNA	fresh soil-forest- depth20cm-replicate-3	GATCGA
F4-20	16S rRNA	fresh soil-forest- depth20cm-replicate-4	CGCGCG
F1-25	16S rRNA	fresh soil-forest- depth25cm-replicate-1	AGCTGA
F2-25	16S rRNA	fresh soil-forest- depth25cm-replicate-2	CACAGT
F3-25	16S rRNA	fresh soil-forest- depth25cm-replicate-3	GTACAC
G1-0	16S rRNA	fresh soil-grass- depth0cm-replicate-1	GTGTGT
G2-0	16S rRNA	fresh soil-grass- depth0cm-replicate-2	GTGTGT
G3-0	16S rRNA	fresh soil-grass- depth0cm-replicate-3	ATGCTA
G4-0	16S rRNA	fresh soil-grass- depth0cm-replicate-4	TCGAGA
G1-5	16S rRNA	fresh soil-grass- depth5cm-replicate-1	GACTAG
G2-5	16S rRNA	fresh soil-grass- depth5cm-replicate-2	TACGTA
G3-5	16S rRNA	fresh soil-grass- depth5cm-replicate-3	CACAGT
G4-5	16S rRNA	fresh soil-grass- depth5cm-replicate-4	TCTCTC
G1-10	16S rRNA	fresh soil-grass- depth10cm-replicate-1	GAGATC
G2-10	16S rRNA	fresh soil-grass- depth10cm-replicate-2	TAGCAT
G3-10	16S rRNA	fresh soil-grass- depth10cm-replicate-3	CAGTCA
G4-10	16S rRNA	fresh soil-grass- depth10cm-replicate-4	ACACGT
G1-15	16S rRNA	fresh soil-grass- depth15cm-replicate-1	GATCGA
G2-15	16S rRNA	fresh soil-grass- depth15cm-replicate-2	TATACG
G3-15	16S rRNA	fresh soil-grass- depth15cm-replicate-3	CATGAC
G4-15	16S rRNA	fresh soil-grass- depth15cm-replicate-4	ACGTAC
G1-20	16S rRNA	fresh soil-grass- depth20cm-replicate-1	GTACAC
G2-20	16S rRNA	fresh soil-grass- depth20cm-replicate-2	TCAGAG
G3-20	16S rRNA	fresh soil-grass- depth20cm-replicate-3	CGATAT
G4-20	16S rRNA	fresh soil-grass- depth20cm-replicate-4	ACTGCA
G1-25	16S rRNA	fresh soil-grass- depth25cm-replicate-1	GTCACA
G2-25	16S rRNA	fresh soil-grass- depth25cm-replicate-2	TCGAGA
G3-25	16S rRNA	fresh soil-grass- depth25cm-replicate-3	CGCGCG
G4-25	16S rRNA	fresh soil-grass- depth25cm-replicate-4	AGAGTC
pmoA-B1-0	*pmoA*	fresh soil-bog- depth0cm-replicate-1	CACAGT
pmoA-B2-0	*pmoA*	fresh soil-bog- depth0cm-replicate-2	GACTAG
pmoA-B3-0	*pmoA*	fresh soil-bog- depth0cm-replicate-3	TACGTA
pmoA-B1-5	*pmoA*	fresh soil-bog- depth5cm-replicate-1	CAGTCA
pmoA-B2-5	*pmoA*	fresh soil-bog- depth5cm-replicate-2	GAGATC
pmoA-B3-5	*pmoA*	fresh soil-bog- depth5cm-replicate-3	TAGCAT
pmoA-B1-10	*pmoA*	fresh soil-bog- depth10cm-replicate-1	CATGAC
pmoA-B2-10	*pmoA*	fresh soil-bog- depth10cm-replicate-2	GATCGA
pmoA-B3-10	*pmoA*	fresh soil-bog- depth10cm-replicate-3	TATACG
pmoA-B1-15	*pmoA*	fresh soil-bog- depth15cm-replicate-1	CGATAT
pmoA-B2-15	*pmoA*	fresh soil-bog- depth15cm-replicate-2	GTACAC
pmoA-B3-15	*pmoA*	fresh soil-bog- depth15cm-replicate-3	TCAGAG
pmoA-B1-20	*pmoA*	fresh soil-bog- depth20cm-replicate-1	CGCGCG
pmoA-B2-20	*pmoA*	fresh soil-bog- depth20cm-replicate-2	GTCACA
pmoA-B3-20	*pmoA*	fresh soil-bog- depth20cm-replicate-3	TCGAGA
pmoA-B1-25	*pmoA*	fresh soil-bog- depth25cm-replicate-1	CGTATA
pmoA-B2-25	*pmoA*	fresh soil-bog- depth25cm-replicate-2	GTGTGT

pmoA-B3-25	*pmoA*	fresh soil-bog- depth25cm-replicate-3	TCTCTC
pmoA-F1-0	*pmoA*	fresh soil-forest- depth0cm-replicate-1	ACACGT
pmoA-F2-0	*pmoA*	fresh soil-forest- depth0cm-replicate-2	ATATCG
pmoA-F3-0	*pmoA*	fresh soil-forest- depth0cm-replicate-3	CGATAT
pmoA-F1-5	*pmoA*	fresh soil-forest- depth5cm-replicate-1	ACGTAC
pmoA-F2-5	*pmoA*	fresh soil-forest- depth5cm-replicate-2	ATCGAT
pmoA-F3-5	*pmoA*	fresh soil-forest- depth5cm-replicate-3	CGCGCG
pmoA-F1-10	*pmoA*	fresh soil-forest- depth10cm-replicate-1	ACTGCA
pmoA-F2-10	*pmoA*	fresh soil-forest- depth10cm-replicate-2	ATGCTA
pmoA-F3-10	*pmoA*	fresh soil-forest- depth10cm-replicate-3	CGTATA
pmoA-F1-15	*pmoA*	fresh soil-forest- depth15cm-replicate-1	AGAGTC
pmoA-F2-15	*pmoA*	fresh soil-forest- depth15cm-replicate-2	CACAGT
pmoA-F3-15	*pmoA*	fresh soil-forest- depth15cm-replicate-3	GACTAG
pmoA-F1-20	*pmoA*	fresh soil-forest- depth20cm-replicate-1	AGCTGA
pmoA-F2-20	*pmoA*	fresh soil-forest- depth20cm-replicate-2	CAGTCA
pmoA-F3-20	*pmoA*	fresh soil-forest- depth20cm-replicate-3	GAGATC
pmoA-F1-25	*pmoA*	fresh soil-forest- depth25cm-replicate-1	AGTCAG
pmoA-F2-25	*pmoA*	fresh soil-forest- depth25cm-replicate-2	CATGAC
pmoA-F3-25	*pmoA*	fresh soil-forest- depth25cm-replicate-3	GATCGA
pmoA-G1-0	*pmoA*	fresh soil-grass- depth0cm-replicate-1	GTACAC
pmoA-G2-0	*pmoA*	fresh soil-grass- depth0cm-replicate-2	TCAGAG
pmoA-G3-0	*pmoA*	fresh soil-grass- depth0cm-replicate-3	AGAGTC
pmoA-G1-5	*pmoA*	fresh soil-grass- depth5cm-replicate-1	GTCACA
pmoA-G2-5	*pmoA*	fresh soil-grass- depth5cm-replicate-2	TCGAGA
pmoA-G3-5	*pmoA*	fresh soil-grass- depth5cm-replicate-3	AGCTGA
pmoA-G1-10	*pmoA*	fresh soil-grass- depth10cm-replicate-1	GTGTGT
pmoA-G2-10	*pmoA*	fresh soil-grass- depth10cm-replicate-2	TCTCTC
pmoA-G3-10	*pmoA*	fresh soil-grass- depth10cm-replicate-3	AGTCAG
pmoA-G1-15	*pmoA*	fresh soil-grass- depth15cm-replicate-1	TACGTA
pmoA-G2-15	*pmoA*	fresh soil-grass- depth15cm-replicate-2	ACACGT
pmoA-G3-15	*pmoA*	fresh soil-grass- depth15cm-replicate-3	ATATCG
pmoA-G1-20	*pmoA*	fresh soil-grass- depth20cm-replicate-1	TAGCAT
pmoA-G2-20	*pmoA*	fresh soil-grass- depth20cm-replicate-2	ACGTAC
pmoA-G3-20	*pmoA*	fresh soil-grass- depth20cm-replicate-3	ATCGAT
pmoA-G1-25	*pmoA*	fresh soil-grass- depth25cm-replicate-1	TATACG
pmoA-G2-25	*pmoA*	fresh soil-grass- depth25cm-replicate-2	ACTGCA
pmoA-G3-25	*pmoA*	fresh soil-grass- depth25cm-replicate-3	ATGCTA

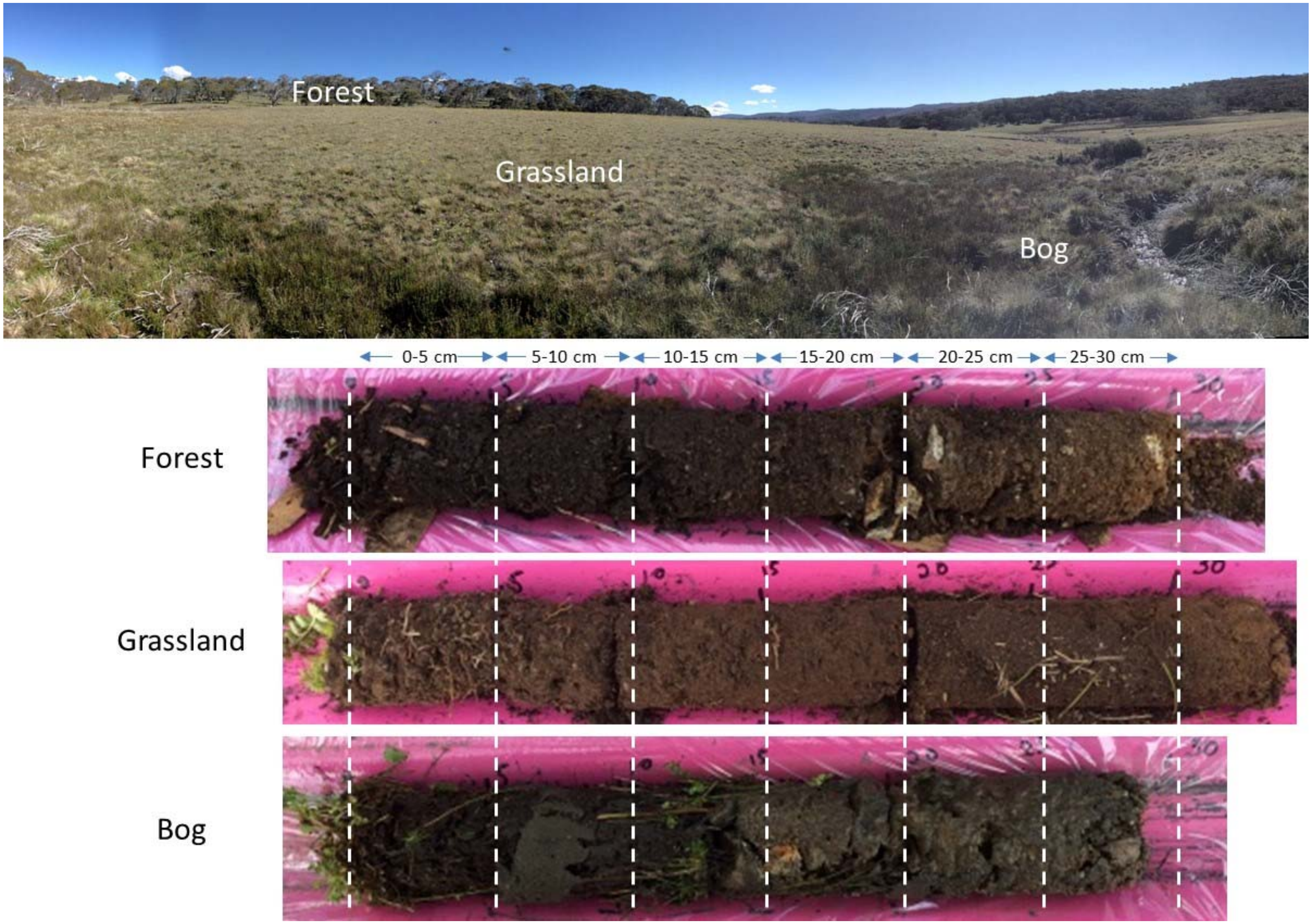

Figure S1. Soil/Vegetation gradient (Forest-Grassland-Bog) located in Snowy Mountains region near Kosciuszko National Park, NSW, Australia. Soil/vegetation types shown above, below are three soil cores from each of the soil/vegetation types.

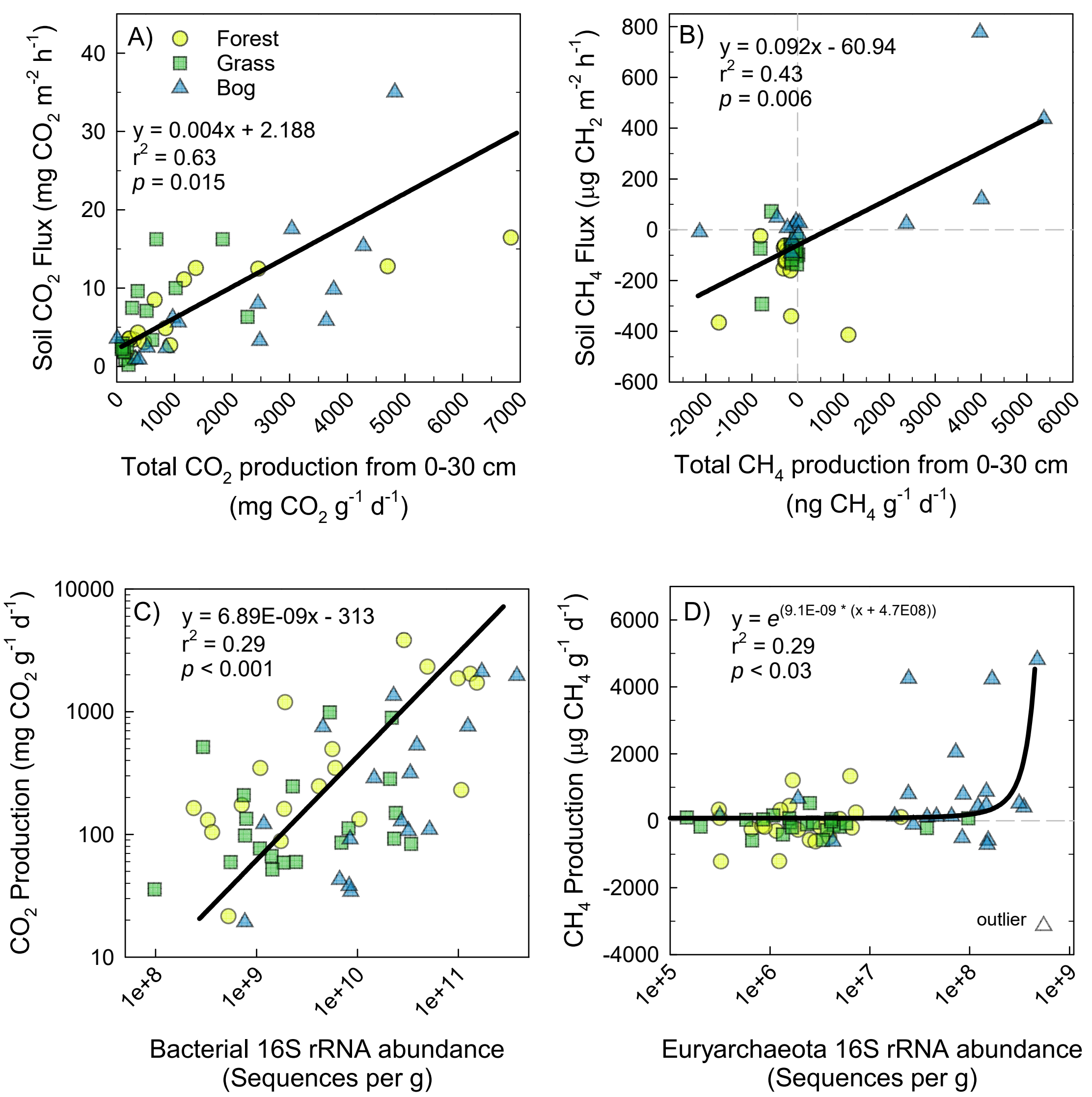

Figure S2. Correlations between total CO_2 production and CO_2 soil-atmosphere flux (**A**), CH_4 production and CH_4 soil-atmosphere flux (**B**). Total GHG production was calculated for each soil profile as sum of production from 0 to 30 cm depth. Regressions between bacterial 16S rRNA abundance with CO_2 production (**C**, linear equation with log, log scale). Regression between Euryarchaeota 16S rRNA and CH_4 production (**D**, 3-parameter exponential, log x-axis). Euryarchaeota abundances were calculated by multiplying the archaeal 16S rRNA qPCR values with the proportion of the archaeal 16S rRNA reads assigned to Euryarchaeota. Equations, chosen by best fit, are shown in each panel.

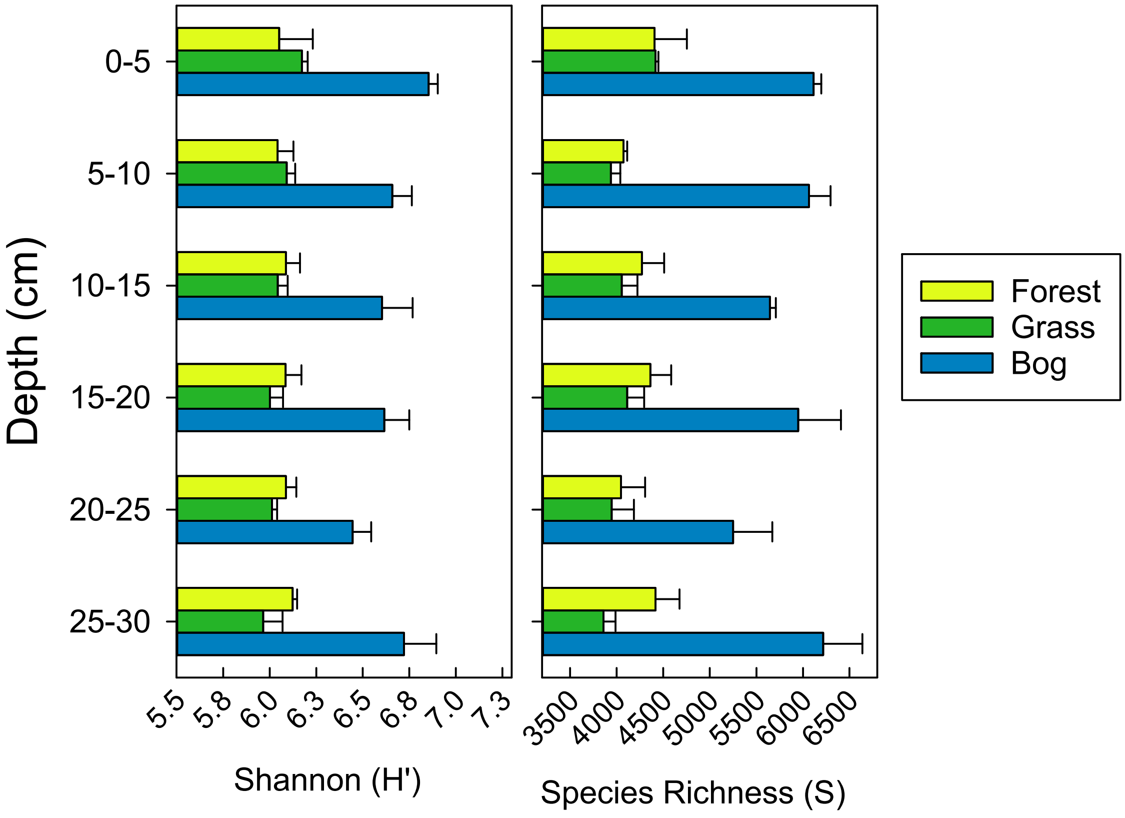

Figure S3. Bacterial 16S rRNA gene diversity measured as Shannon Diversity (H') and Richness (S) (n=4, means ± standard errors).

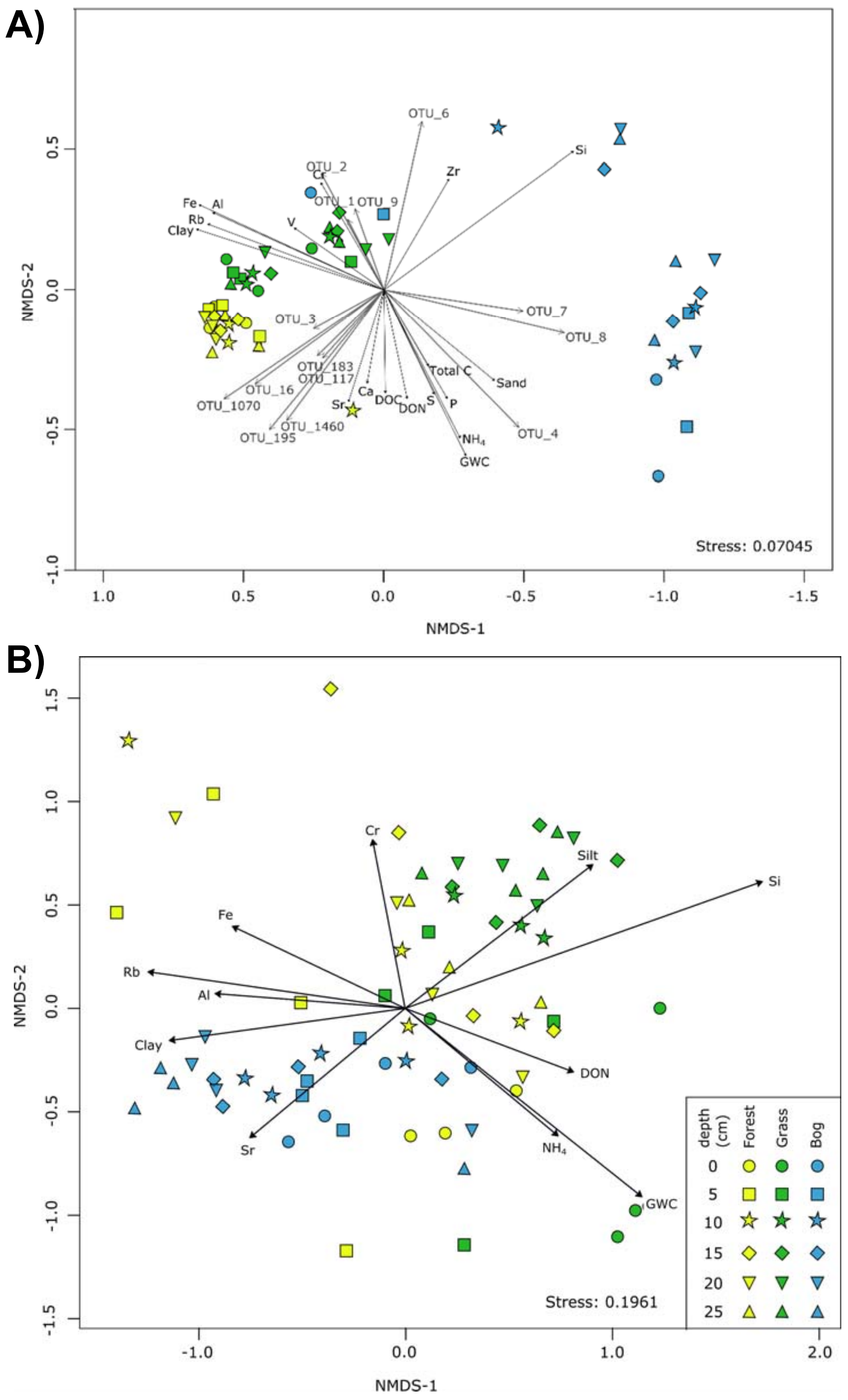

Figure S4. NMDS ordination of *pmoA* (A) and euryarchaeota (B) communities based on the Bray–Curtis dissimilarity of community composition. Shape indicates depth and sites are colored according to the soil type. The arrows indicate the direction at which the environmental vectors fit the best (using the *envfit* function) onto the NMDS ordination space. Abbreviations: DOC, dissolved organic carbon; DON, dissolved organic nitrogen; EC, electrical conductivity; GWC, gravimetric water content; NH_4, ammonium.

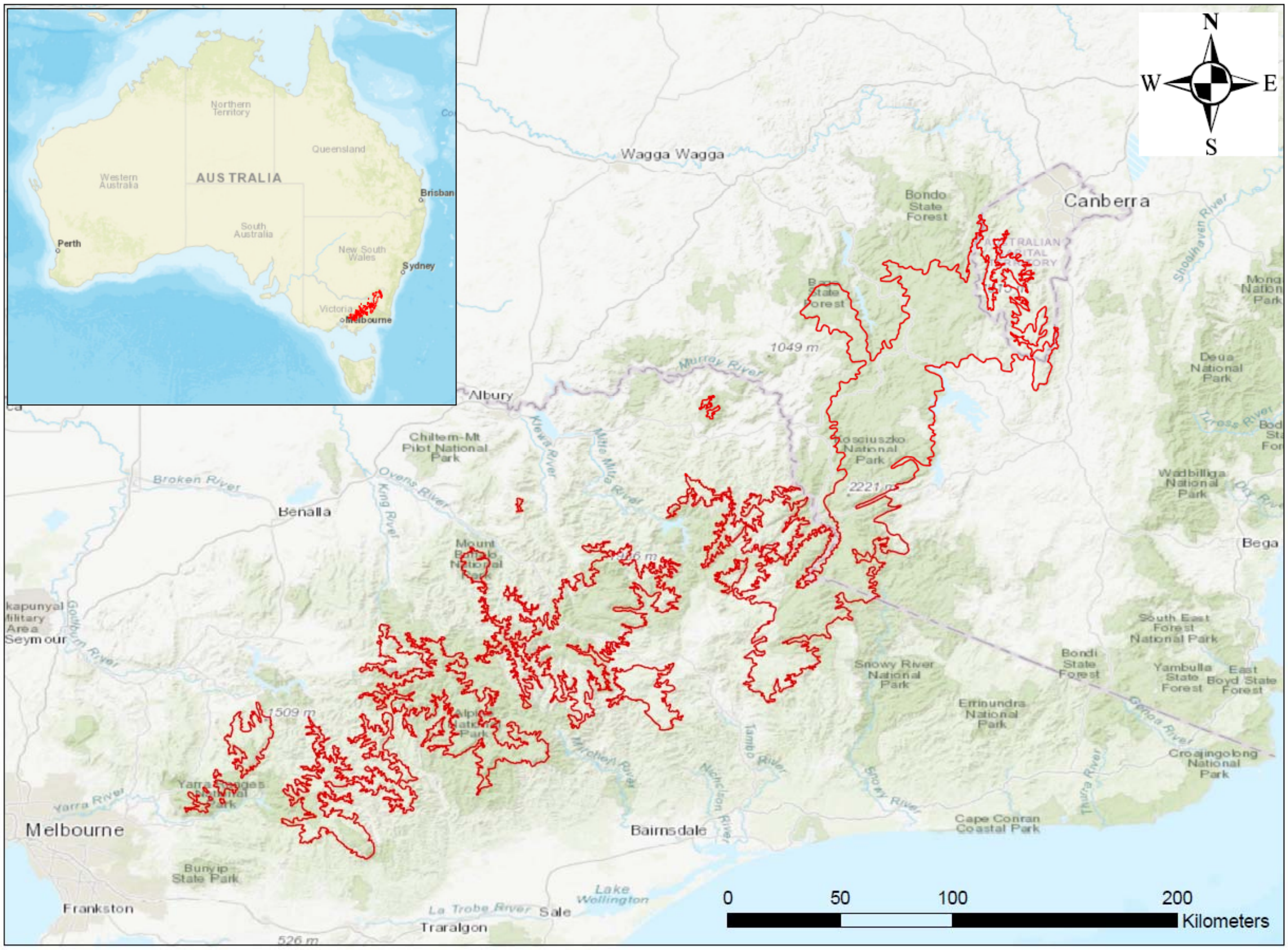

Figure S5. Areal coverage of Australian Alps in southeastern Australia (1.2M ha).
